# Mediterranean Diet and Agri-Food By-Products: A Possible Sustainable Approach for Breast Cancer Treatment

**DOI:** 10.3390/antiox14070789

**Published:** 2025-06-26

**Authors:** Pasquale Perrone, Chiara De Rosa, Stefania D’Angelo

**Affiliations:** 1 Department of Medical, Movement, and Wellbeing Sciences, Parthenope University of Naples, 80133 Naples, Italy; pasquale.perrone@collaboratore.uniparthenope.it; 2 School of Medicine and Surgery, Department of Clinical Medicine and Surgery, University of Naples Federico II, 80131 Naples, Italy; chiara.derosa8@studenti.unina.it

**Keywords:** Mediterranean diet, polyphenols, breast cancer, phytochemicals, nutrition, sustainability

## Abstract

The sustainable use of agri-food by-products offers a significant opportunity. Increasing evidence shows that these by-products have various bioactive compounds that help reduce inflammation and, in turn, the severity of several proliferative diseases. Numerous epidemiological studies have suggested an inverse relationship between the consumption of fruits and vegetables and the incidence of breast cancer. Anti-breast cancer effects involve a variety of mechanisms, inhibiting proliferation, migration, metastasis, and angiogenesis of breast tumor cells; inducing apoptosis and cell cycle arrest; and enhancing the sensitivity of breast tumor cells to radiotherapy and chemotherapy. Extensive research suggests that the Mediterranean diet has various bioactive compounds known to provide protective effects against a wide range of non-communicable diseases. Among the phytochemicals identified as protective against breast cancer, natural polyphenols have shown antioxidant, anti-inflammatory, immunomodulatory, and anticancer properties. This review highlights the potential role of natural dietary products and their primary bioactive components in preventing and treating breast cancer, with special emphasis on the mechanisms of action. The integration of agri-food by-products into the diet not only offers opportunities for the prevention and treatment of breast cancer but also promotes sustainable use of resources, contributing to the reduction of waste and the improvement of global health.

## 1. Introduction

Breast cancer is among the most prevalent types of cancer and remains a leading cause of cancer-related mortality in women worldwide [1]. According to global statistics, in 2020 breast cancer accounted for over 2.26 million new cases, making it the most diagnosed cancer that year. Although in 2022 the number of new cases increased to over 2.31 million, breast cancer became the second most common cancer globally, following lung cancer. Despite this shift, breast cancer continues to be the most diagnosed cancer in women and the primary cause of cancer death among females. Overall, it ranks as the fourth most common cause of cancer-related death across both sexes [2].

The development of breast cancer is multifactorial, involving an interplay between genetic, environmental, and lifestyle-related factors. Although genetic predisposition, including mutations in BRCA1 and BRCA2, is a significant risk factor, these hereditary mutations account for only 5–10% of all breast cancer cases [3].

Many breast cancers are sporadic and are attributed to modifiable lifestyle, reproductive, and environmental exposures. Exogenous risk factors include early menarche, nulliparity, oral contraceptive use, limited or absent breastfeeding, use of hormone replacement therapy, alcohol consumption, diabetes, obesity, and circadian rhythm disruption such as night-shift work [4,5]. These factors can contribute to hormonal imbalances, inflammation, and metabolic dysfunction, creating a cellular environment conducive to tumorigenesis.

Factors like high breast density and increasing age are natural risk contributors. At the same time, behaviors such as alcohol consumption, smoking, poor diet, physical inactivity, and circadian rhythm disruption have been linked to increased breast cancer incidence. These influences can lead to oxidative stress, excessive production of free radicals, hormonal imbalances, and chronic inflammation, all of which promote tumor initiation and progression [6,7].

At the molecular level, breast cancer is highly heterogeneous. It is commonly classified based on the presence or absence of specific receptors: estrogen receptor (ER), progesterone receptor (PR), and human epidermal growth factor receptor 2 (HER2). This classification defines several molecular subtypes with distinct biological behaviors and therapeutic responses. ER-positive subtypes, such as those represented by MCF-7 and T47D cell lines, generally respond to hormone therapies. ER-negative subtypes, including cell lines like MDA-MB-231, MDA-MB-468, SKBR3, and MDA-MB-453, often require alternative treatment strategies due to their resistance to hormone-based therapies [8].

Further stratification includes luminal A, luminal B, HER2-enriched, and basal-like subtypes. Of clinical interest is triple-negative breast cancer (TNBC), which lacks expression of ER, PR, and HER2. TNBC often overlaps with the basal-like subtype and is characterized by aggressive behavior, poor prognosis, and limited treatment options. The therapeutic response differs markedly among these subtypes, making personalized treatment approaches essential and highlighting the complexity of managing breast cancer [9,10]. Understanding these distinctions is crucial not only for improving therapeutic strategies but also for developing targeted prevention and early detection methods aimed at reducing the global burden of breast cancer.

Since most breast cancers are non-hereditary, prevention efforts must focus on addressing modifiable risk factors and understanding molecular mechanisms beyond genetics. Notably, approximately 30% of sporadic breast cancer cases show epigenetic alterations, especially changes in DNA methylation, suggesting that epigenetic modulation could serve as a valuable target for future therapies [11].

Given that breast cancer is the most common cancer among women and the second most often occurring cancer overall worldwide, the identification and development of new, more effective preventive and therapeutic strategies are of critical importance [12]. Currently, the standard treatment options for early-stage breast cancer include surgical resection, adjuvant chemotherapy, radiotherapy, and hormone therapy. While these strategies have contributed to improved survival rates, their long-term effectiveness is limited by the development of drug resistance and the emergence of severe side effects [13]. Moreover, certain subtypes of breast cancer, such as TNBC, do not respond to hormone therapy, making them especially challenging to treat. These limitations underscore the urgent need for more effective, targeted, and less toxic approaches to breast cancer prevention and treatment.

Traditional breast cancer therapies, while widely used, are associated with several drawbacks, including poor long-term outcomes, adverse side effects, and the high risk of metastasis, recurrence, and resistance to treatment. In response, researchers have turned their attention to alternative therapeutic strategies, particularly those derived from natural sources. Since ancient times, plant-based and non-herbal remedies have been used to treat various ailments. Today, this historical knowledge is being reexamined through the lens of modern science, leading to increased interest in nutraceuticals, especially phytochemicals, for their potential anticancer properties [11].

Reflecting this trend, the global nutraceuticals market, valued at approximately $79 billion in 2017, is projected to grow dramatically, reaching around $734 billion by 2026 [14,15]. Phytochemicals offer promising advantages due to their lower toxicity, ability to target multiple molecular pathways, and potential to complement conventional therapies. They are also being explored as adjuvant agents that can reduce chemotherapy’s adverse effects and resistance.

### Agri-Food By-Products as a Potential Target for the Treatment of Breast Cancer

The World Cancer Research Fund underscores the pivotal role of diet in the prevention of various diseases, including cancer. Extensive research shows that a diet abundant in vegetables, fruits, whole grains, and legumes is associated with a reduced risk of chronic illnesses, thereby promoting overall health [16]. Conversely, insufficient consumption of these food groups has been correlated with the onset and progression of many diseases. Studies estimate that approximately 20% of cancers could be prevented through a diet rich in diverse plant-based foods. Epidemiological data on cancer incidence support this premise, as lower cancer rates are seen in regions such as Southern Europe and Southeast Asia compared to more industrialized areas like Northern Europe and North America. This disparity suggests that, in addition to genetic and environmental factors, diet is a fundamental part of disease prevention [17].

In 2010, the Food and Agriculture Organization (FAO) introduced the concept of “sustainable diets,” defined as dietary patterns that minimize environmental impact while ensuring food security, nutrition, and long-term health. However, food waste remains a pressing global concern, with nearly one-third of all food produced being discarded. This wastage is not only an economic and environmental challenge but also a missed opportunity, as many food by-products have bioactive compounds with significant potential applications. Rather than being disposed of, these by-products, including peels, seeds, shells, pomace, and leaves, can be integrated into a circular economy to recover valuable nutrients and bioactive substances [18].

Agri-food by-products have garnered increasing attention due to their potential applications in the production of functional foods, nutraceuticals, and food additives [19]. These by-products can be classified based on their chemical structure, botanical origin, or biological activity. These by-products are rich in bioactive compounds such as polyphenols, antioxidants, anthocyanins, carotenoids, fatty acids, peptides, dietary fibers, and enzymes [20,21]. Beyond the food industry, these compounds are also of considerable interest in pharmaceutical and cosmetic applications.

Agri-food by-products have increasingly gained attention due to their potential role in preventing oxidative damage and alleviating inflammatory conditions [22]. Among the various bioactive components derived from plant sources, phytochemicals have appeared as key contributors to these health benefits, thanks to their potent antioxidant, anti-aging, anti-inflammatory, and antiproliferative activities.

## 2. Phytochemicals: Naturally Occurring Compounds in the Human Diet

**Figure 1:**
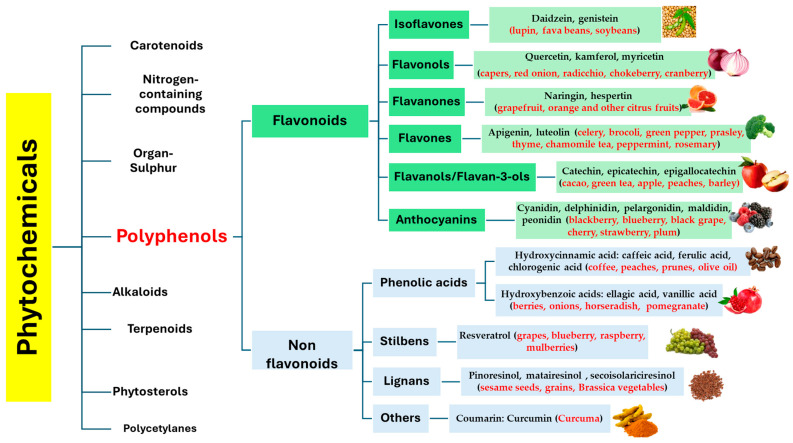
Classification of phytochemicals.

Phytochemicals are bioactive non-nutrient compounds widely distributed in plants that can decrease the risk of various syndromes [19]. Numerous databases have been developed to catalog dietary phytochemicals, providing information on their occurrence, bioavailability, and health-promoting effects [23,24] (Figure 1). To date, researchers have found more than 5000 distinct phytochemicals in foods such as fruits, vegetables, cereals, and legumes [24], although it is believed that many more remain undiscovered.

Unlike essential nutrients, these compounds are not needed for basic human survival, yet their presence in the diet is associated with a range of protective effects. An expanding body of scientific evidence suggests that phytochemicals interact synergistically with nutrients, vitamins, minerals, and dietary fiber found in fruits, vegetables, grains, and legumes, enhancing the overall protective potential of plant-based diets. Several studies have proven their anti-carcinogenic and antimutagenic properties, showing that regular consumption of phytochemicals may significantly reduce the risk of developing chronic diseases, including various types of cancer [25,26,27,28,29,30].

Phytochemicals, naturally occurring compounds in the human diet, have gained attention for their role in breast cancer prevention and treatment due to their abundance, low toxicity, and diverse mechanisms of action. These include tannins, flavonoids, organic acids, and alkaloids. Compounds such as genistein, curcumin, resveratrol, and epigallocatechin gallate have shown the ability to inhibit tumor growth by affecting cellular signaling pathways, estrogen biosynthesis enzymes, and processes like proliferation, invasion, and angiogenesis. Some act as phytoestrogens, influencing various receptors and epigenetic factors. Additionally, certain phytochemicals enhance the effectiveness of chemotherapeutic drugs by inhibiting drug resistance-related transport proteins and targeting breast cancer stem cells. However, their effects can vary with concentration, and clinical efficacy is limited by issues related to bioavailability and tissue distribution. Further research is needed to better understand their metabolism and absorption in the human body [31].

Also, many plant parts, which are not otherwise consumed, for example, the peel or the pericarp of certain fruits, have similar potent phytochemicals that can be isolated and translated into marketable drugs. A few of the studies have pointed to the relevance of these phytochemicals in overcoming multidrug resistance, which is a limiting factor in drug-based cancer treatments [32]. A great deal of research, therefore, is currently focused on finding alternatives that have minimal side effects but lend themselves to use in highly effective targeted or combinatorial therapies. *Punica granatum*, or pomegranate, is one such potential candidate. The plant has been shown, in several studies, to have phytochemicals that target multiple compounds involved in various steps of carcinogenesis, making them potential candidates for use in targeted breast cancer therapies [33,34,35].

Chemically, phytochemicals are commonly categorized into major groups, including phenolics, carotenoids, alkaloids, nitrogen-containing compounds, and organosulfur compounds (Figure 1). Among these, phenolic compounds, also referred to as polyphenols, are the most extensively studied due to their wide distribution in plant foods and their broad spectrum of biological activities.

In summary, phytochemicals, especially polyphenols, represent a valuable group of compounds with significant implications for public health. Their incorporation into daily diets, particularly through the consumption of whole, minimally processed plant-based foods, may offer substantial benefits in the prevention of chronic diseases and the maintenance of overall health and well-being.

## 3. Polyphenols: The Most Abundant Phytochemicals in the Plant Kingdom

Polyphenols are the most abundant phytochemicals in the plant kingdom, and they are particularly concentrated in fruits, vegetables, and certain plant-based beverages such as tea, coffee, and red wine. They are non-nutrient secondary metabolites naturally synthesized by plants, commonly present in fruits, vegetables, seeds, and nuts that are part of the daily human diet. The estimated number of PPs present in the human diet in developed countries exceeds 10,000 compounds, with concentrations varying depending on factors such as sun exposure, rainfall, degree of ripeness, post-harvest processing, storage conditions, and cooking techniques [36,37]. In nature, PPs serve specific functions: they attract pollinating insects, protect plants from various threats (UV radiation, insects, fungi, diseases, etc.), and provide them with an “appetizing” color. These compounds handle various sensory properties of foods, such as taste, aroma, color, and astringency. For example, the bitterness of dark chocolate and the astringency of red wine are directly linked to their polyphenol content. From a health perspective, PPs have gained significant attention due to their antioxidant, anti-inflammatory, and anti-carcinogenic properties. They are known to neutralize free radicals, modulate enzyme activities, influence cell signaling pathways, and protect against chronic diseases, including cardiovascular diseases, diabetes, and certain types of cancer. They have been studied for their potential to slow the progression of degenerative diseases and are increasingly being explored as therapeutic agents, particularly in the context of breast cancer [38,39,40].

### 3.1. Polyphenols Classification

Polyphenols are classified according to the number of phenol rings in their structure and the types of elements or functional groups to which these rings are bound. Their basic chemical structure generally features one or more phenolic rings linked to various substituents such as sugars, amines, carboxylic and organic acids, lipids, or added phenolic units. These structural differences influence their solubility, bioavailability, and biological activity. The main polyphenol classes are broadly grouped into two main categories: flavonoids and non-flavonoids. The flavonoid group includes six principal subclasses: flavonols, flavones, flavanones, flavanols, isoflavones, and anthocyanins, while the non-flavonoid group encompasses phenolic acids, lignans, stilbenes, and tannins (Figure 1) [41].

Today, PPs are recognized as one of the most extensively studied classes of secondary plant metabolites, appreciated for their wide-ranging bioactivities and roles in plant physiology and human health. Despite their structural heterogeneity, they share key physicochemical properties, most notably, their ability to reduce oxidative stress, chelate metal ions, and form complexes with proteins. These characteristics underpin both their biological effects and practical applications. Furthermore, PPs contribute to the color, flavor, and nutritional quality of plant-derived foods, making them essential not only for plant defense and reproduction but also for the organoleptic and functional properties of human diets.

These natural compounds play a crucial role in mitigating oxidative stress-related disorders, including aging [21,42,43,44], cardiovascular diseases [22,45], neurodegenerative conditions [46,47], type 2 diabetes [48,49,50], and various forms of cancer [22,23,24,25,26,27,28,29].

Particularly significant within the class of PPs are flavonoids, phenolic acids, and tannins. These compounds exert a variety of metabolic effects: they modulate carbohydrate and lipid metabolism, reduce hyperglycemia and dyslipidemia, improve insulin sensitivity, support pancreatic β-cell function and adipose tissue regulation, and promote insulin secretion. Furthermore, they are known to counteract oxidative stress [51,52,53] and inhibit stress-responsive signaling pathways and inflammatory processes, highlighting their potential in the prevention and management of metabolic and inflammatory diseases [54,55,56,57].

PPs have been studied for their potential in slowing the progression of degenerative diseases and are increasingly being explored as therapeutic agents, especially in the context of breast cancer [37,38]. Among the many biological activities attributed to PPs, their antioxidant and anti-inflammatory properties are the most consistently documented and widely studied [21,52,58,59,60,61,62,63,64]. These properties are particularly relevant in the context of aging and inflammation. Reactive oxygen species (ROS), often produced by senescent cells (SCs) as part of the senescence-associated secretory phenotype (SASP), play a central role in promoting systemic inflammation [65,66]. In this context, the ability of PPs to scavenge ROS and modulate inflammatory signaling pathways supports their classification as anti-SASP agents, potentially contributing to the reduction of age-related chronic inflammation.

### 3.2. Oxidative Stress and Inflammation Processes

Oxidative stress occurs when there is an imbalance between the production of ROS and the body’s ability to eliminate them [67]. ROS originate from enzymatic reactions (phagocytosis, mitochondrial respiratory chain, prostaglandin synthesis) and non-enzymatic reactions (spontaneous oxidation, radiation exposure) [68]. Exogenous sources include radiation, pollution, and cigarette smoke, while endogenous sources involve enzymatic systems such as NADPH oxidase, cyclooxygenase, and lipoxygenase [69]. Therefore, ROS-induced damage to DNA, proteins, and lipids is assessed instead. Since ROS production varies by tissue, it is difficult to prove absolute concentration levels for physiological and pathological conditions [70]. At low concentrations, ROS play beneficial roles (cell synthesis, immune defense), but excessively, they contribute to cardiovascular, neurological, renal, and rheumatic diseases [71]. The body counters ROS damage with enzymatic antioxidants (SOD, CAT, GPx) and non-enzymatic antioxidants (glutathione, vitamins A, C, E) SOD reduces O_2_^−^ to H_2_O_2_, preventing further radical formation, while CAT and GPx convert H_2_O_2_ into water and oxygen [71].

Reactive nitrogen species (RNS) also result from metabolism and contribute to various diseases (diabetes, Parkinson’s, cardiovascular, and neurological disorders). Nitric oxide (NO) is produced from arginine breakdown and has antimicrobial, vasodilatory, and cell signaling functions [72]. However, NO interacts with O_2_^−^ to form peroxynitrite, which damages endothelial cells, oxidizes DNA, and leads to lipid peroxidation.

Although ROS and RNS are known for their damaging effects, at low levels, they function as regulatory mediators in cellular signaling. The threshold at which their effects shift from beneficial to harmful is still unclear. An imbalance between increased ROS/RNS production and reduced antioxidant defense leads to chronic inflammation, triggering pro-inflammatory gene expression and activating factors such as Nf-κB, which enhances cytokine synthesis and immune cell recruitment [73]. Inflammation also activates enzymes like cyclooxygenase and lipoxygenase, leading to prostaglandin and leukotriene synthesis.

Inflammation can amplify oxidative stress: neutrophils and macrophages generate ROS and RNS to eliminate pathogens, but excessive immune responses cause these species to damage surrounding tissues. Since oxidative stress and inflammation are tightly interconnected, their prevention and control may offer effective strategies for managing chronic diseases [73,74]. Inflammation is a physiological defense mechanism activated in response to infections or trauma, aiming to find the pathological agent, digest it, and restore homeostasis. While it is a protective response, uncontrolled inflammation can lead to acute and chronic diseases [75]. If unresolved, it can develop into chronic inflammation, with a prolonged immune response lasting months to years, resulting in significant healthcare costs. Inflammation is triggered by infectious agents or trauma, activating both innate and adaptive immunity. Pathogen recognition starts a cascade of biological reactions mediated by pro-inflammatory compounds, such as cytokines and chemokines [76], which recruit immune cells and promote vasodilation to help their migration. Neutrophils, the key cells of innate immunity, release toxic compounds like proteases and reactive oxygen and nitrogen species to destroy pathogens [77]. Once the pathogen is eliminated, monocytes are recruited to promote wound healing and block further inflammatory processes. Chronic inflammation is linked to diseases such as atherosclerosis, diabetes, metabolic disorders, cancer, and autoimmune conditions, making it crucial to prevent its progression.

### 3.3. Anti-Breast Cancer Activity of Polyphenols

Polyphenols exert their cancer-preventive effects by modulating growth factor signaling pathways. Growth factors are integral to cellular processes such as proliferation, differentiation, and tissue repair. However, dysregulation of these pathways, resulting from genetic mutations, receptor overexpression, or other factors, can lead to uncontrolled cellular proliferation, a hallmark of cancer. A key target in oncological research is the EGFR, which plays a crucial role in cell proliferation and survival. EGFR has been extensively studied for its involvement in tumorigenesis, making it a focal point for the development of anticancer therapies. Additionally, EGFR is implicated in various inflammatory conditions, including thrombin-mediated inflammation, virus-induced respiratory inflammation, cardiac inflammation, neuroinflammation, and dermatological disorders. It is also associated with the progression of malignancies such as liver, gastrointestinal, breast, lung, and prostate cancers [78,79].

Chronic inflammation is a critical factor in cancer progression, as prolonged inflammatory states contribute to tumor development by increasing oxidative stress and inducing DNA damage. Inflammatory cells within the tumor microenvironment promote genomic instability, angiogenesis, and overall tumor progression. Consequently, anti-inflammatory compounds hold significant potential in interfering with early cancer progression and mitigating malignancy [80].

Emerging evidence suggests that dietary PPs can modulate EGFR-mediated signaling and influence the inflammatory milieu of tumors. These natural compounds not only attenuate the aggressiveness of cancer cells but also show cytotoxic properties at elevated concentrations, positioning them as promising candidates for cancer therapy. This review examines the dual role of polyphenolic derivatives and their prospective applications in human health, with a specific focus on their therapeutic potential in breast cancer treatment. By leveraging agri-food by-products, researchers aim to develop innovative and sustainable strategies for disease prevention and treatment, ultimately fostering more efficient use of natural resources [81].

PPs combat oxidative stress, which triggers various transcriptional pathways, such as those regulated by nuclear factor erythroid 2–related factor 2 (Nrf2), nuclear factor κB (NF-κB), activator protein-1 (AP-1), peroxisome proliferator-activated receptor-γ (PPAR-γ), p53, hypoxia-inducible factor-1α (HIF-1α), and the Wnt/β-catenin system. These pathways mediate the expression of over 500 genes involved in growth factors and their receptors, cell cycle regulation, and inflammatory cytokines, contributing to chronic inflammation, cancer, diabetes, neurodegeneration, as well as cardiovascular and pulmonary diseases [15,82,83]. PPs’ anticancer properties include inhibiting tumor transformation, cell growth, invasion, metastasis, and angiogenesis while promoting apoptosis [84,85,86].

One of the key mechanisms by which PPs act against breast cancer is their antioxidant ability. They neutralize ROS and reduce oxidative stress, which is a major factor in DNA damage and cancer development. By enhancing the body’s natural antioxidant defenses, such as superoxide dismutase and catalase, polyphenols help protect healthy cells while making cancer cells more vulnerable to stress-induced death [87]. Another important mechanism is the induction of apoptosis, or programmed cell death, in cancer cells. Polyphenols trigger apoptotic pathways by activating proteins such as p53 and caspases while downregulating anti-apoptotic proteins like Bcl-2 and Bcl-xL. This leads to mitochondrial dysfunction and the release of cytochrome c, which ultimately causes cancer cells to die without harming surrounding healthy cells [88].

PPs also inhibit cancer cell proliferation by interfering with the cell cycle. They suppress the activity of key regulators like cyclin D1 and cyclin-dependent kinases, which handle cancer cell division. By arresting cells in specific phases of the cycle, PPs slow down tumor growth and prevent the spread of malignant cells. Furthermore, these compounds can block angiogenesis, the process by which tumors develop new blood vessels to sustain their growth. By inhibiting vascular endothelial growth factor (VEGF) and other pro-angiogenic factors, PPs starve cancer cells of the nutrients and oxygen they need to thrive.

In addition to these effects, PPs also modulate various signaling pathways involved in cancer progression. For instance, they can inhibit the NF-κB and Wnt/β-catenin pathways, which are commonly activated in breast cancer cells. By disrupting these signaling cascades, PPs reduce inflammation, limit tumor invasion, and enhance the body’s ability to suppress cancer growth.

## 4. The Foods of the Mediterranean Diet: A Polyphenolic-Food Strategy Against Breast Cancer

The term “Mediterranean diet” was popularized by Ancel Keys, an American physiologist who saw the dietary patterns of populations in Southern Italy and correlated them with low rates of chronic diseases and longer life expectancy [89]. The Mediterranean diet (MedDiet) is a dietary pattern traditionally followed by populations living in countries bordering the Mediterranean Sea, particularly Southern Europe [90,91]. It is primarily based on the frequent intake of plant-based foods such as vegetables, fruits, legumes, whole grains, and nuts, and moderate consumption of dairy products and fish. Olive oil is the predominant source of dietary fat, and red wine is occasionally consumed in moderate amounts during meals. This diet is not uniform but reflects regional agricultural traditions and cultural variations across different Mediterranean countries. Nonetheless, its common nutritional principles have been associated with many health benefits [90]. Over time, this dietary model has gained international recognition, including from institutions such as UNESCO, the World Health Organization (WHO), and the Food and Agriculture Organization (FAO).

Several studies have shown a strong association between adherence to the MedDiet and a reduced incidence of various chronic diseases, including cardiovascular disease, type 2 diabetes, obesity, and certain cancers, particularly breast cancer. The protective role of this diet against breast cancer has been linked to its high content of dietary antioxidants, anti-inflammatory agents, and PPs, which act through multiple biological mechanisms including modulation of oxidative stress, hormone metabolism, and immune responses [92].

Dietary PPs, abundantly found in fruits, vegetables, olive oil, nuts, and red wine, may influence signaling pathways related to inflammation and cellular proliferation. These include the NF-κB, MAPK, and PI3K/Akt pathways. Moreover, PPs enhance the activity of antioxidant enzymes such as SOD, catalase, and glutathione peroxidase (GPx), thereby contributing to cellular protection against oxidative damage. The traditional use of wild and seasonal plants in Mediterranean regions adds further nutritional and therapeutic value. Many of these plants, such as wild greens, pomegranates, citrus fruits, and red onions, are rich in antioxidant phytochemicals that help mitigate the effects of ROS. Their inclusion in the diet may contribute to the observed lower rates of metabolic and cardiovascular diseases in Mediterranean populations [93].

Overall, the MedDiet offers a holistic nutritional approach that integrates healthy eating habits with lifestyle practices, such as regular physical activity and social mealtime traditions. Its adoption has shown promise not only in promoting general well-being but also in reducing the risk and progression of breast cancer and other chronic conditions [94].

The MedDiet is widely recognized for its protective effects against chronic diseases, including certain types of cancer [95]. Among its key components, many plant-based foods are rich in PPs, bioactive compounds known for their antioxidant, anti-inflammatory, and anticancer properties.

The agri-food by-products may be used as co-chemotherapeutic agents to improve the pharmacological action of anticancer drugs in breast cancer management and improve the disease-associated inflammatory status. Studies have been carried out by combining natural compounds with approved drugs. As already specified, these compounds might also exert pro-oxidant activity. The following section outlines the anti-breast cancer properties of several polyphenol-rich foods commonly found in the MedDiet [96]. These include extra virgin olive oil, berries, citrus fruits, and nuts, all of which contribute to cellular protection through mechanisms such as oxidative stress reduction, cell cycle regulation, and induction of apoptosis.

### 4.1. Fruits

Fruits are rich in bioactive compounds extracted from various parts of the plant, used to treat a range of medical disorders. Fruits are typically rich in PPs, which contribute to their strong antioxidant properties and may play a role in lowering cancer risk [97,98]. A meta-analysis of fifteen prospective studies found that a high intake of fruits was associated with a modest reduction in the risk of breast cancer (summary RR for highest vs. lowest intake: 0.92; 95% CI = 0.86–0.98; I^2^ = 9%) [99,100]. Additionally, another meta-analysis showed a borderline inverse relationship between pre-diagnostic fruit consumption and overall survival in breast cancer patients (summary HR for highest vs. lowest intake: 0.83; 95% CI = 0.67–1.02; I^2^ = 0%) [101]. Certain fruits, such as pomegranate, mangosteen, and citrus fruits, have proven inhibitory effects on breast cancer cells.

#### 4.1.1. *Punica granatum* L.

Commonly known as pomegranate, it is a fruit-bearing plant of the *Lythraceae* family, originally native to Persia and now extensively cultivated in India, Iran, the Caucasus, and throughout the Mediterranean region. Historically valued for its medicinal properties, pomegranate is now recognized in modern biomedical research for its potent antioxidant, anti-inflammatory, and anticancer activities, primarily due to its high content of PPs, especially ellagitannins [43,102,103,104].

Numerous preclinical studies have shown that pomegranate extracts show significant anticancer effects, particularly in breast cancer models. The fruit’s bioactive constituents, including anthocyanins (delphinidin, cyanidin, pelargonidin), hydrolyzable tannins (punicalagin, gallagic acid), flavonoids (kaempferol, quercetin, luteolin), and conjugated fatty acids such as punicic acid, play a role in modulating diverse biological pathways relevant to cancer progression [33,105].

Pomegranate extracts, including juice, seed oil, and peel extract, have been shown to inhibit key processes in breast cancer pathogenesis, such as cell proliferation, angiogenesis, and metastasis, while promoting apoptosis. In vitro studies on estrogen receptor-positive (MCF-7) and estrogen receptor-negative (MDA-MB-231) breast cancer cell lines have proven that PPs derived from fermented juice exert stronger antiproliferative effects than those from fresh juice. Pomegranate seed oil has shown up to 90% inhibition of proliferation and 75% reduction in cell invasion in MCF-7 cells, along with a 54% increase in apoptosis in MDA-MB-435 metastatic cells [106]. Additionally, fermented juice PPs and seed oil were reported to downregulate VEGF, a central proangiogenic factor, and upregulate migration-inhibitory factor (MIF), further supporting their anticancer activity [107].

Further investigations have shown that pomegranate extract can induce cell cycle arrest at both G2/M and G0/G1 phases in MCF-7 and MDA-MB-231 cells and promote apoptosis through suppression of estrogen receptor and Wnt/β-catenin signaling pathways [108,109,110]. Pomegranate peel extract also significantly modulated apoptotic markers by increasing Bax and decreasing Bcl-2 expression [111].

Importantly, pomegranate has also proved potential for chemoprevention of hormone-dependent breast cancers through its action as a phytoestrogen. The plant’s extracts inhibit aromatase, the key enzyme in estrogen biosynthesis, thereby decreasing estradiol levels and preventing estradiol-induced proliferation in estrogen-responsive breast cancer cells [106,107,108,109,110,111,112]. Moreover, pericarp extracts act as selective estrogen receptor modulators, capable of binding estrogen receptors without promoting uterine proliferation.

In addition to its phytoestrogenic and antiproliferative effects, pomegranate also exerts anti-inflammatory actions by inhibiting cyclooxygenase enzymes, particularly COX-2, which is often upregulated in breast cancer. By reducing prostaglandin E2 production, pomegranate extracts may contribute to a decline in both tumor progression and estrogen synthesis [113].

Metastasis, a major challenge in breast cancer treatment, has also been shown to be suppressed by pomegranate. Seed oil and whole-fruit extracts significantly inhibited cell migration and invasion by downregulating RhoA and RhoC, modulating the NF-κB signaling pathway, and influencing SP1 and HMMR expression [106,114]. The antiangiogenic potential of pomegranate has been proved through its capacity to downregulate VEGF, VEGFR, and survivin, and to upregulate antiangiogenic factors like MIF [112,115]. Pomegranate was also found to suppress IL-8, a chemokine involved in angiogenesis, suggesting a multitargeted approach in disrupting neovascularization.

Finally, evidence from organ culture and in vivo models supports the chemopreventive activity of pomegranate. In murine mammary gland cultures, both fermented juice and seed oil significantly inhibited DMBA-induced tumorigenesis [106,116].

Overall, the anticancer activity of *Punica granatum* in breast cancer is mediated through multiple mechanisms, including regulation of cell proliferation, hormonal signaling, apoptosis, angiogenesis, and metastasis. Although these preclinical findings are promising, clinical trials and pharmacokinetic studies are needed to fully elucidate the therapeutic potential and safety profile of pomegranate-derived products in human subjects.

#### 4.1.2. Apples

Apples, (*Malus* spp., Rosaceae), a staple of the MedDiet, are a nutritious and versatile fruit enjoyed across the region. Rich in fiber, vitamins, and antioxidants, they support heart health and aid digestion. In Mediterranean cuisine, apples are consumed fresh, baked, or added to salads and desserts, making them a delicious and healthy choice for daily consumption. They are a notable source of phytochemicals, especially flavonoids, and have high concentrations of PPs and other health-promoting compounds [117].

Apples are among the most consumed fruits and contribute significantly to the human diet. Several clinical studies have investigated the effects of PPs found in apples and apple-based products, such as juices, reporting encouraging health outcomes [118]. For example, eating at least one apple per day has been associated with a reduced risk of colorectal cancer (odds ratio = 0.65; 95% confidence interval, 0.39–1.09) [119,120,121].

The *Annurca* apple (*Malus pumila* cv *Annurca*), a variety native to Campania, Italy, is recognized for its high content of PPs, bioactive compounds known for their antioxidant, anti-inflammatory, and anticancer properties. Some studies conducted have explored the effects of these PPs on breast cancer, showing promising therapeutic potential. A study examined the antiproliferative activity of *Annurca* apple polyphenol extract (AAPE) on human breast cancer cells (MCF-7). The results showed that AAPE strongly inhibited cell proliferation by inducing a G2/M phase cell cycle arrest and promoting apoptosis. Immunoblot analysis revealed increased levels of p53 and p21, reduced expression of cyclin D1, and inhibition of ERK1/2 phosphorylation. Additionally, AAPE treatment significantly increased the pro-apoptotic Bax/Bcl-2 ratio, along with the cleavage of caspases-9, -6, -7, and poly (ADP-ribose) polymerase (PARP) [121]. Another study focused on the effects of AAPE on TNBC cells (MDA-MB-231). The results proved that AAPE selectively inhibited cell viability, induced a G2/M cell cycle arrest associated with increased p27 and phosphorylated cdc25C, and reduced p21. AAPE also promoted the generation of ROS in MDA-MB-231 cells, while acting as an antioxidant in non-tumor MCF10A cells. ROS generation was identified as the primary mechanism of the antitumor activity of AAPE, triggering sustained activation of the JNK pathway and inhibition of cell growth and survival [55].

Studies suggest that PPs from *Annurca* apples, through mechanisms such as oxidative stress induction, cell cycle arrest, and apoptosis activation, may have beneficial effects in the prevention and treatment of breast cancer. However, further research is needed to confirm these findings and evaluate the clinical efficacy of these compounds. These studies show that the PPs found in *Annurca* apples may offer promising anticancer properties, particularly in breast cancer prevention and therapy. However, additional clinical trials are needed to fully understand their therapeutic potential [57].

Flavonoids isolated from both the peel and flesh of the *Pink Lady* apple cultivar have shown inhibitory activity against MCF-7 breast cancer cells, with half-maximal inhibitory concentrations (IC_50_) reported at 58.42 ± 1.39 mg/mL and 296.06 ± 3.71 mg/mL, respectively [122]. Similarly, research on the *Pelingo* apple variety revealed that its juice, rich in polyphenolic compounds, suppressed the proliferation of MCF-7 and MDA-MB-231 breast cancer cell lines. The juice also impeded tumor promotion induced by 12-O-tetradecanoylphorbol-13-acetate in pre-neoplastic cells, by reducing colony formation and blocking TPA-stimulated ERK1/2 phosphorylation [123].

Phloretin, a polyphenol found in apples, has been proven to inhibit the growth and migration of TNBC cells, thus helping prevent metastasis. Phloretin has also been reported to increase the anticancer effects of chemotherapy drugs like Adriamycin (doxorubicin), Taxol (paclitaxel), and cisplatin, as well as potentiating the treatment effects of tamoxifen and Herceptin. Furthermore, phloretin has been shown to reverse multidrug resistance in cancer cells [124].

A comprehensive review highlighted that apple PPs, such as flavonoids and phenolic acids, have been associated with reduced cancer risk through various mechanisms, including antioxidant activity, modulation of cell signaling pathways, and regulation of gene expression. Data suggest that regular consumption of apples and apple-derived products may contribute to cancer prevention strategies.

#### 4.1.3. Citrus Fruits

Citrus fruits, including oranges, lemons, grapefruits, mandarins, and limes, are among the most widely consumed fruits worldwide. Citrus fruits are characteristic of the Mediterranean region, even though their botanical origin is in Asia (India, China, and Southeast Asia). However, they have spread and adapted perfectly to the Mediterranean basin thanks to its mild climate, becoming an integral part of the local diet, agricultural culture, and culinary tradition [125]. Citrus fruits are an important source of bioactive phytochemicals, particularly PPs, flavonoids, and limonoids, which contribute to their health-promoting properties. The most prominent PPs include flavanones such as naringenin, hesperidin, eriocitrin, and diosmin, which are well known for their antioxidant, anti-inflammatory, and potentially anticancer effects. In addition to flavonoids, citrus fruits also have phenolic acids (e.g., ferulic acid and caffeic acid), carotenoids (such as beta-cryptoxanthin), and essential oils rich in terpenes like limonene, which has demonstrated chemopreventive activity in experimental breast cancer models. The peel and seeds of citrus fruits, often considered by-products, are particularly rich in these compounds. Regular consumption of citrus or citrus-derived extracts has been associated with reduced oxidative stress, improved immune responses, and the modulation of metabolic pathways involved in cancer development [126,127].

Rich in vitamin C, flavonoids, carotenoids, and limonoids, these fruits offer many health benefits, notably their potential protective effects against several forms of cancer, including breast cancer. Emerging research has focused on the bioactive compounds in citrus fruits and their mechanisms of action in reducing breast cancer risk and modulating tumor progression.

Citrus fruits are especially rich in flavonoids such as hesperidin, naringenin, tangeretin, and nobiletin. These compounds have antioxidant, anti-inflammatory, antiproliferative, and pro-apoptotic properties. Studies have proven that these PPs influence various molecular pathways involved in carcinogenesis, including inhibition of cell proliferation, induction of apoptosis, and suppression of angiogenesis and metastasis [128,129].

Naringenin, a flavanone found abundantly in grapefruits and oranges, has been shown to exert cytotoxic effects on breast cancer cells by modulating key cell signaling pathways such as the PI3K/Akt, NF-κB, and MAPK pathways. In MCF-7 breast cancer cells, naringenin has been proven to induce G0/G1 cell cycle arrest and downregulate cyclin D1 expression, ultimately reducing cell proliferation [130].

Similarly, hesperidin, primarily found in oranges, shows both antiproliferative and pro-apoptotic properties. In vitro studies have revealed that hesperidin can activate caspase-3 and -9, key enzymes in the apoptotic cascade, and reduce oxidative stress by enhancing antioxidant enzyme activity [125,131]. The inhibition of ROS-induced DNA damage by hesperidin is particularly relevant in the context of estrogen-mediated breast carcinogenesis, where oxidative stress plays a key role.

The antitumor activity of citrus-derived compounds is attributed to several complementary mechanisms. Firstly, their antioxidant ability neutralizes ROS, preventing oxidative DNA damage and mutation accumulation, a hallmark of cancer development [132]. Secondly, citrus flavonoids show estrogenic and anti-estrogenic activities, which are especially important in hormone-dependent breast cancers. Naringenin, for instance, can bind estrogen receptors and modulate their activity, making it a potential natural selective estrogen receptor modulator [133].

In addition to direct effects on tumor cells, citrus PPs modulate the tumor microenvironment. Tangeretin and nobiletin, polymethoxylated flavones found in tangerines, inhibit angiogenesis by downregulating VEGF expression, thereby restricting tumor blood supply and growth [134]. These compounds also reduce the expression of matrix metalloproteinases, enzymes involved in extracellular matrix degradation and tumor metastasis [135].

Numerous in vitro and animal studies have provided promising evidence of the anticancer potential of citrus fruits. For example, a study using nude mice implanted with human breast cancer cells showed that oral administration of nobiletin significantly inhibited tumor growth and angiogenesis [136]. Similarly, hesperidin and tangeretin were shown to suppress breast cancer cell migration and invasion, suggesting their utility in preventing metastasis.

Epidemiological data further support a protective role. A meta-analysis of observational studies showed that higher citrus fruit consumption was associated with a reduced risk of breast cancer, particularly among postmenopausal women [99,100]. Although human clinical trials are limited, some studies report that diets enriched with citrus bioactives can improve markers of oxidative stress and inflammation in patients at high risk of cancer, suggesting a chemopreventive effect.

An emerging area of interest is the potential of citrus flavonoids to enhance the efficacy of existing chemotherapeutic agents while minimizing their side effects. Preclinical studies have shown that naringenin can sensitize breast cancer cells to doxorubicin and paclitaxel, two commonly used anticancer drugs, through the modulation of drug resistance pathways [137]. This suggests that citrus compounds may be used as adjuvants in breast cancer therapy to improve outcomes and reduce toxicity.

Moreover, citrus flavonoids may offer protection against chemotherapy-induced oxidative damage in normal tissues. By reducing systemic oxidative stress and inflammation, they could improve patient tolerance to treatment and reduce the risk of secondary complications such as cardiotoxicity. The biological activities of naringenin, hesperidin, tangeretin, and nobiletin include the modulation of oxidative stress, inhibition of cell proliferation and angiogenesis, and enhancement of apoptosis [138].

While preclinical evidence is robust, more clinical studies are needed to fully prove their role in breast cancer prevention and treatment. Nonetheless, their integration into a balanced, plant-rich diet such as MedDiet appears to be a safe and promising strategy for reducing breast cancer risk.

#### 4.1.4. Walnuts

Walnuts (*Juglans regia*) are a notable source of bioactive nutrients, including polyunsaturated fatty acids, fiber, vitamins, minerals, and especially PPs. These phenolic compounds, mainly concentrated in the seed coat, include ellagic acid, ellagitannins, catechins, and flavonoids. Walnut PPs show strong antioxidant and anti-inflammatory activities, and several studies have suggested their potential role in breast cancer prevention and therapy [139].

Ellagic acid has been shown to promote apoptosis and suppress proliferation in breast cancer cells, as proved in both in vitro experiments and animal models. Experimental research has reported that walnut-enriched diets can reduce tumor incidence and growth in mouse models of mammary cancer, likely through the modulation of inflammation-related signaling pathways, cell proliferation, and estrogen response [140,141].

In a clinical trial, women with breast cancer consumed 2 ounces of walnuts daily for approximately two weeks between their diagnostic biopsy and later surgery. RNA sequencing of tumor samples revealed significant changes in the expression of 456 genes. These changes were associated with the activation of pathways promoting apoptosis and cell adhesion, and the inhibition of pathways related to cell proliferation and migration. The study suggests that walnut consumption could suppress the growth and survival of breast cancer in humans [142].

The synergistic interaction of walnut components, healthy fats, PPs, and phytosterols may provide cellular protection against oxidative stress and carcinogenic mechanisms. Therefore, regular and moderate consumption of walnuts may serve as an effective dietary strategy for reducing breast cancer risk, particularly when included as part of a MedDiet [143].

#### 4.1.5. Berries

In the MedDiet, berries, such as strawberries, blueberries, raspberries, and blackberries, are highly valued for their rich nutritional profile and health benefits. These fruits are abundant in antioxidants, particularly anthocyanins, which help reduce inflammation and lower the risk of heart disease. Additionally, their high fiber content supports digestive health and contributes to overall wellness. Other fruits commonly consumed in the Mediterranean region include grapes and figs, both of which are integral to the local diet and culinary traditions [144]. The PPs found in berries, such as anthocyanins, flavonoids (quercetin, kaempferol), ellagic acid, tannins, resveratrol, and catechins, work synergistically to offer a broad range of health benefits. These PPs are potent antioxidants that help combat oxidative stress, reduce inflammation, and protect against chronic conditions such as heart disease, cancer, and neurodegenerative disorders. Including berries in the MedDiet contributes significantly to overall health and wellness. Several types of berries, besides grapes, have been shown to have potential anticancer effects on breast cancer cells. For instance, methanolic extracts from strawberries showed cytotoxic properties against T47D breast cancer cells in vitro and significantly reduced tumor growth in a murine breast adenocarcinoma model by promoting apoptotic pathways [145]. Similarly, bilberry extract was shown to suppress the proliferation of MCF-7 cells in a dose-dependent fashion, with an IC_50_ ranging between 0.3 and 0.4 mg/mL, and this effect was accompanied by the induction of apoptosis [146]. Moreover, cranberry extract was also found to inhibit MCF-7 cell proliferation, a result associated with the activation of apoptotic mechanisms and cell cycle arrest at the G1 phase [147]. Animal and cell-based studies have shown that extracts from berries, particularly blueberries, blackberries, and strawberries, can inhibit the growth of breast cancer cells. These studies suggest that the PPs in berries may affect signaling pathways involved in cancer cell proliferation, apoptosis, and metastasis [148]. Human studies are more limited but promising. Some observational studies have found that a higher intake of fruits and vegetables, including berries, is associated with a lower risk of breast cancer. However, more clinical trials are needed to confirm the specific effects of berries on breast cancer prevention or treatment [149,150].

### 4.2. Extra Virgin Olive Oil

One of the core components of the MedDiet is extra virgin olive oil (EVOO), which is rich in monounsaturated fatty acids (mainly oleic acid) and bioactive compounds such as phenolic antioxidants, hydroxytyrosol, oleuropein, and tyrosol. These components are believed to contribute significantly to the protective effects of the diet against oxidative stress, inflammation, and metabolic disorders. Squalene and other minor constituents in EVOO have shown promising biological activities [151,152,153,154,155,156,157]. EVOO is a fundamental element of MedDiet. As the principal source of fat in this dietary pattern, EVOO consumption has been linked to a decreased incidence of several chronic conditions, including breast cancer [158]. Although the precise molecular mechanisms by which dietary lipids influence the development of breast cancer are not yet fully elucidated, accumulating evidence points to multiple biological actions of EVOO. These include changes to cellular membrane composition, modulation of gene expression, interference with intracellular signaling pathways, reductions in oxidative damage, and improvements in immune response [159,160]. EVOO is notably rich in phenolic compounds; over 30 have been found. Among them are flavonoids such as luteolin and various phenolic acids, including caffeic, gallic, *p*-coumaric, vanillic, and ferulic acids. Luteolin has proved the ability to induce apoptosis in triple-negative breast cancer cells (ER-/PR-/HER2-), as well as to suppress angiogenesis and inhibit aromatase activity, the latter being involved in the conversion of androgens to estrogens. The oil also has potent bioactive compounds like oleuropein, hydroxytyrosol, and oleocanthal. These PPs have pronounced antioxidant, anti-inflammatory, and anticancer effects. Oleuropein and its metabolite hydroxytyrosol have been shown to reduce tumor cell proliferation and promote apoptosis by modulating oxidative status, metabolic processes, and transcriptional activity. Oleocanthal has displayed selective cytotoxicity against cancer cells without affecting normal cells and has also been shown to potentiate the action of targeted therapies like trastuzumab in HER2-positive breast cancer cases [161,162]. Further, in vitro research has revealed that EVOO PPs can induce arrest of the cell cycle at the G2/M phase, inhibit proteins that prevent apoptosis, and reduce the expression of HER2, an oncogene commonly overexpressed in aggressive breast cancer subtypes [151]. These properties show a possible synergistic effect with conventional chemotherapy.

Population studies support the beneficial role of EVOO. A large Spanish cohort study involving 4282 women found that consuming two or more tablespoons of EVOO per day was associated with a 68% decrease in the risk of developing malignant breast cancer [163]. Similar associations have been seen in epidemiological studies across Sweden, Italy, Greece, and the Canary Islands, where high olive oil intake correlated with a lower breast cancer risk [153,164]. In addition to its antioxidant effects, EVOO exhibits anti-inflammatory activity comparable to that of nonsteroidal drugs such as ibuprofen, which is significant given the well-established link between chronic inflammation and cancer development [165]. Experimental studies have further shown that EVOO high in oleocanthal content can selectively eliminate malignant cells while sparing healthy tissue, underscoring their potential for cancer therapy [165,166]. While a systematic review and meta-analysis conducted by Sealy and colleagues assessed the connection between olive oil consumption and breast cancer risk across ten observational studies, the results did not reach statistical significance, and the overall quality of evidence was rated as very low [152]. Although further clinical trials are needed to confirm these findings, incorporating EVOO into a balanced dietary regimen is a promising preventive strategy [167].

### 4.3. Flaxseeds

Flaxseeds, which have been consumed for thousands of years, are small brown or golden seeds rich in dietary fiber, omega-3 fats, and lignans. Their estrogenic activity comes from lignans metabolizing into enterodiol and enterolactone in the digestive tract. Compared to soy products, flax seeds have stronger phytoestrogens and significantly affect 2-hydroxyestrone elimination [168]. In one study, mice were given a lignan-rich diet after being induced with cancer, leading to a reduction in tumor load [169]. Flax seeds and secoisolariciresinol diglycoside helped decrease malignancies. In another experiment, human breast cancer cells were injected into mice, followed by an 8-week basal diet. One group received a 10% flaxseed diet, reducing cancer growth by 45% [170]. Additionally, flax seeds improved mammary gland morphogenesis in female mice, increasing terminal end buds, ducts, and epithelial cell division. Mice with a 10% flaxseed diet showed lower breast tumor incidence after carcinogen exposure [171]. Overall, flax seeds promote mammary tissue differentiation, prevent malignancies, and reduce tumor development, making female offspring less vulnerable to carcinogens.

### 4.4. Red Wine

Red wine, a common part of the MedDiet, has attracted significant interest in its potential health-promoting effects, largely due to its richness in polyphenolic compounds. Notable among these are resveratrol, quercetin, and catechins, which are recognized for their antioxidant, anti-inflammatory, and chemopreventive actions. Numerous experimental studies have examined the connection between moderate red wine intake and breast cancer risk, resulting in findings that, while complex, are promising [172,173,174,175].

Resveratrol, a polyphenol, is naturally present in grapes, red wine, certain berries, oilseeds like peanuts, and specific plants. In grapes, it is concentrated in the skin, and its concentration in wine is influenced by factors such as grape variety, cultivation region, and duration of fermentation [176]. This compound is known for its potent antioxidant and anti-inflammatory properties [177], and it plays a pivotal role in regulating apoptosis [178,179].

In vitro studies have highlighted resveratrol’s ability to counteract cancer by impeding the proliferation of various breast cancer cell lines, such as MCF-7 and MDA-MB-231. It achieves this through mechanisms including the induction of apoptosis, modulation of estrogen receptor pathways, and disruption of the cell cycle [180,181]. Additionally, resveratrol has been shown to decrease HER2 expression, a receptor often overexpressed in aggressive breast cancer types [182].

Further research has proved resveratrol’s influence on DNA methylation, affecting genes associated with cancer progression [183]. Stilbenoids like resveratrol can reshape DNA methylation profiles in breast cancer cells and suppress oncogenic NOTCH signaling by epigenetically regulating MAML2 expression [184].

This compound promotes apoptosis through diverse pathways and offers various protective effects on neurological, cardiovascular, and hepatic systems. It has also been reported to combat oxidative stress, viral infections, and the proliferation of cancer cells. Notably, resveratrol impairs breast cancer stem cells (BCSCs), reduces their tumor-forming potential, and inhibits mammosphere formation by targeting the Wnt/β-catenin pathway. Moreover, it affects cancer-associated fibroblasts by downregulating critical oncogenic markers like MMP-9, MMP-2, c-Myc, and Cyclin D1 [185,186].

Preclinical investigations have shown that resveratrol can inhibit breast cancer cell growth by modulating several molecular pathways. For example, in estrogen receptor-positive (ER+) cell lines, it can trigger cell cycle arrest and apoptosis by downregulating cyclin D1 and increasing the expression of p53 and Bax. Moreover, it has been seen to inhibit aromatase activity, potentially lowering estrogen synthesis and thereby the risk of hormone-dependent breast cancers [187].

Despite these benefits, the relationship between red wine consumption and breast cancer remains debated, primarily due to the presence of ethanol. Alcohol, irrespective of its source, has been classified as a Group 1 carcinogen by the International Agency for Research on Cancer (IARC), and many studies have associated its consumption with an increased risk of breast cancer in women [188]. A meta-analysis encompassing over one million women found that every added 10 g of daily alcohol intake correlates with a 7–10% higher risk of breast cancer [189]. Some research attempts to distinguish red wine from other alcoholic beverages, proposing that its polyphenol content might mitigate alcohol’s adverse effects [190]. For example, red wine PPs have been shown to influence estrogen metabolism by inhibiting the enzyme CYP19 (aromatase) and changing the ratio between 2-hydroxyestrone and 16α-hydroxyestrone, metabolites implicated in breast cancer risk [179]. Nonetheless, the health benefits of wine-derived PPs may not be sufficient to counteract the harmful effects of ethanol, especially when consumption exceeds moderate levels. Leading health authorities recommend limiting alcohol consumption to reduce cancer risk, including breast cancer [191]. Epidemiological studies offer a nuanced perspective. Some studies show that moderate red wine intake (up to one glass per day) may correlate with a lower risk of breast cancer, possibly due to its antioxidant content [192]. Others suggest that even minimal alcohol consumption can raise breast cancer risk through mechanisms involving estrogen metabolism and DNA damage [172,193]. Thus, moderation is crucial, and the balance between beneficial compounds and alcohol content plays a central role.

In conclusion, while compounds like resveratrol in red wine show anticancer activity in laboratories and animal models, the ethanol part may negate these advantages in human populations. As such, moderate consumption is essential, and PPs from non-alcoholic sources may present a safer strategy for reducing breast cancer risk.

## 5. The Mediterranean Diet and Breast Cancer: A Protective Nutritional Approach

The MedDiet is recognized to prevent several human diseases, including endothelial damage, dysmetabolism, and cardiopathy. Extracts from edible plants may prevent pathology-associated cell damage, ROS generation, and physiological processes, such as metabolism and inflammation. Consumption of plant products is of great importance in the MedDiet. Wild and in-house edible plants, which are cooked or consumed as raw salads as part of the Mediterranean-style diet, have considerable polyphenolic content and show strong antioxidant activity. In the Mediterranean basin, wild plants rich with antioxidants are harvested and eaten seasonally. For example, red chicory and blueberries, which are vegetables and fruits, are among the richest antioxidants used as traditional food [194].

The MedDiet is considered a sustainable dietary pattern, evaluated through environmental, nutritional, economic, and socio-cultural indicators. However, there is no standardized method for assessing its sustainability. Common indicators include greenhouse gas emissions, water use, and agriculture, while data on nutritional and socio-cultural aspects remains limited. Barriers to adherence include difficulties in making informed food choices, demographic and motivational factors, resistance to change, and socio-cultural influences [195].

**Figure 2:**
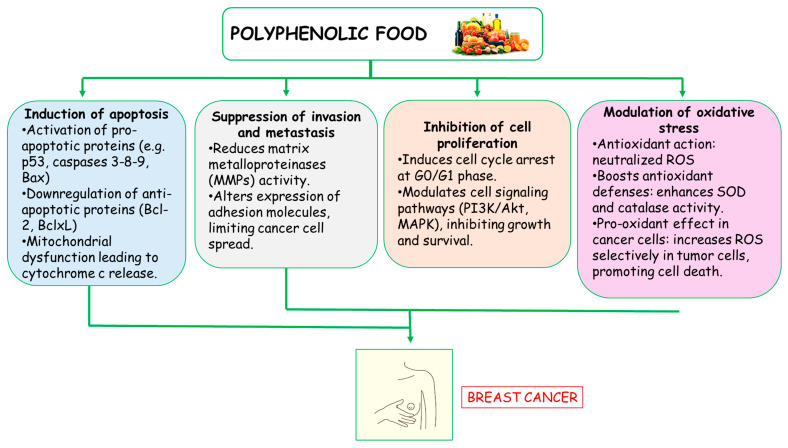
Some molecular effects of dietary polyphenols on breast cancer.

MedDiet is among the most extensively researched dietary patterns, and consistent adherence to it has been linked to a reduced risk of various chronic diseases, including cancer [91,93], and specifically breast cancer [196,197,198]. Notably, the dietary guidelines promoted by the World Cancer Research Fund (WCRF) for cancer prevention align closely with the principles of the MedDiet [199]. Several epidemiological studies and clinical trials suggest that adherence to the Mediterranean diet is associated with a lower risk of breast cancer development and progression [95,163,200,201,202,203,204,205,206,207,208] (Table 1). This protective effect is attributed to the diet’s rich composition of antioxidants, PPs, fiber, healthy fats, and anti-inflammatory compounds, which collectively help regulate cellular processes, reduce oxidative stress, and modulate hormone levels, factors that are closely linked to breast cancer prevention (Figure 2).

Conversely, diets characterized by Western eating patterns have been associated with an increased risk of breast cancer [209,210].

When examining the influence of diet on disease progression, many studies have yielded inconclusive or non-significant outcomes [211,212,213]. A prospective study conducted by Vrieling and colleagues on 2522 postmenopausal women in Germany found that adherence to a healthy dietary pattern was associated with reduced overall mortality (HR 0.74, 95% CI 0.47–1.15, *p*-trend = 0.02) and lower breast cancer recurrence (HR 0.71, 95% CI 0.48–1.06, *p*-trend = 0.02) among patients diagnosed at stages I–IIIa [214].

A meta-analysis of 41 cohort studies involving breast cancer survivors indicated that high consumption of foods associated with high dietary quality, such as fruits, vegetables, legumes, nuts, and whole grains, and low consumption of red meat were correlated with lower mortality rates (RR 0.74, 95% CI 0.60–0.90, based on three studies). Furthermore, general adherence to a high-quality diet was linked to reduced mortality (RR 0.76, 95% CI 0.60–0.95, three studies) [200]. A separate meta-analysis encompassing 56 observational studies also reported a negative association between MedDiet adherence and cancer-related mortality (RR 0.86, 95% CI 0.81–0.91 across 15 studies), as well as overall mortality (RR 0.92, 95% CI 0.87–0.96 across 16 studies) [95]. The effect of MedDiet on breast cancer recurrence timelines is still under investigation, and future interventional studies are needed to clarify the role of MedDiet adherence both before and after treatment in influencing survival outcomes and disease progression [215].

A study published in JAMA Internal Medicine found that women who adhered closely to the MedDiet supplemented with extra virgin olive oil had a 68% lower risk of developing breast cancer compared to those on a low-fat diet. Another study in the European Journal of Cancer Prevention proves that high adherence to the MedDiet is associated with a significant reduction in the incidence of postmenopausal breast cancer [163]. The anti-inflammatory and hormone-regulating properties of this diet may be particularly beneficial for women with estrogen receptor-positive (ER+) and TNBC, as these subtypes are influenced by hormonal and inflammatory pathways.

“Medifoodomics” (Union of Mediterranean, food, and omics, which recalls the science of biological systems) is an emerging interdisciplinary field that explores the intricate relationship between the MedDiet, food bioactive compounds, and their systemic effects on human health. By integrating nutrition science with molecular biology and omics technologies, Medifoodomics investigates how key dietary components, such as PPs, retinoids, and omega-3 PUFAs, modulate biological pathways linked to chronic diseases, including cardiovascular disorders and cancer. This approach provides a deeper understanding of how the MedDiet contributes to disease prevention and therapeutic advancements, offering new perspectives for personalized nutrition and functional food development [201,216].

To promote adherence, the European Commission’s “From Plate to Plate” strategy aims to develop sustainable labeling (Med Index) that incorporates nutritional, environmental, and social factors. Studies confirm that the MedDiet, along with DASH and plant-based diets, has a lower environmental impact and significant health benefits. The concept of the “Planeterranean diet” encourages countries to embrace their own traditional foods to improve dietary habits and combat diet-related diseases. Finding local foods with similar nutritional properties to those of the MedDiet may help address global health challenges [94].

## 6. Conclusions on Sustainable Breast Cancer Therapy

The sustainability of the food system is an increasingly important issue to address, and the transition toward more sustainable food models is an emerging challenge that is becoming ever more complex to tackle. The event aims to discuss some of the key aspects related to food sustainability, the close interconnection between human and planetary health, and, most importantly, the crucial role of food choices in the context of healthy and sustainable dietary patterns.

Expecting a high consumption of cereals, fruit, vegetables, and legumes, the MedDiet needs much less intensive use of natural resources (soil, water) and greenhouse gas emissions than the model based on the consumption of meat and animal fats [217]. *Vitis vinifera* L. grape skin and seeds are clear examples of waste products that are useful as an energy reserve [218].

The use of agri-food by-products in breast cancer treatment aligns with the principles of sustainability and the circular economy. By using food waste rich in bioactive compounds, this approach provides multiple benefits:

- Reduces Food Waste: Repurposing food processing residues into functional nutraceuticals minimizes environmental impact.
- Cost-Effective Cancer Therapy: Agri-food by-products offer an affordable source of anticancer compounds, making them accessible to a broader population.
- Eco-Friendly and Renewable Resource: Unlike synthetic drugs, plant-based bioactive compounds are naturally available and biodegradable.

Agri-food by-products are a novel and sustainable approach to breast cancer treatment. Their rich composition of PPs, flavonoids, carotenoids, dietary fibers, and essential fatty acids offers significant chemoprotective and synergistic effects when combined with conventional therapies. Despite their promise, further research, including clinical trials and bioavailability optimization, is needed to fully integrate these natural compounds into mainstream cancer treatment. By harnessing the potential of food industry by-products, we can move toward a more sustainable, eco-friendly, and effective approach to breast cancer management, helping both human health and the environment.

The incorporation of natural chemicals into conventional treatments marks a significant advancement in modern healthcare. Many natural medicines, once limited to traditional use, are now undergoing rigorous scientific testing for their effectiveness in treating various medical conditions. This reflects a growing trend toward holistic and integrative therapeutic approaches. However, balancing scientific validation with traditional knowledge is essential for this transition.

The clinical success of compounds like paclitaxel shows the potential of natural substances to complement or even enhance conventional medical treatments. However, their inclusion in standard treatment plans depends on strict clinical validation to ensure their efficacy and safety, whether used alongside or as alternatives to synthetic drugs. While natural compounds often show lower toxicity and greater tolerance, thorough scientific studies and clinical trials are necessary to confirm their safety and effectiveness. The ongoing evaluation and integration of these substances highlight the increasing recognition of their potential benefits in modern medicine.

Research on the recovery of agri-food by-products has grown significantly in recent years. The food industry generates many by-products rich in bioactive compounds that are typically discarded but can serve as valuable raw materials for drug production. Sustainable extraction methods, such as hydroalcoholic extraction for flavonoids like hesperidin, nobiletin, and tangeretin, and hydro-distillation for citrus peel, enable the recovery of these compounds. Due to their antibacterial, antifungal, and anti-inflammatory properties, agri-food waste can also enhance drug bioavailability. Moreover, the low cost of these materials presents an attractive possibility for affordable drug production, particularly in low-income countries where access to healthcare and cancer treatment is limited.

These bioactive compounds also play a role in the development of functional foods and nutraceuticals. Some, like citrus peel extracts, can be used in their natural form, while others, such as tangeretin and quercetin, require modification and nanotechnological encapsulation to improve their stability and solubility. Importantly, natural substances derived from agri-food waste have proved the ability to interfere with cancer cell growth by targeting various biological mechanisms. This suggests potential applications in both cancer prevention, by reducing inflammation linked to tumor development, and in cancer treatment, due to their cytotoxic effects.

However, challenges remain about their clinical application. The bioavailability of certain plant compounds is low, requiring strategies such as nanoparticle formulations or chemical modifications to improve their absorption and therapeutic efficacy. Additionally, further clinical trials are needed to prove standardized dosages and confirm their safety and efficacy in human patients. So, further research is needed to confirm their efficacy, decide the best concentrations, and evaluate their potential as standalone or combination therapies. The limited number of clinical studies underscores the need for added experimental animal research and human trials.

## Figures and Tables

**Table 1:** Key components of the Mediterranean Diet and their role in breast cancer prevention [95,163,200,201,202,203,204,205,206,207,208].

**Extra Virgin Olive Oil: A Powerful Antioxidant Source**. Olive oil, particularly extra virgin olive oil (EVOO), is a fundamental part of the Mediterranean diet and plays a significant role in cancer prevention. It is rich in monounsaturated fatty acids (MUFAs) and polyphenols, such as hydroxytyrosol and oleocanthal, which have potent anti-inflammatory, antioxidant, and anti-proliferative properties. Studies suggest that these bioactive compounds help protect DNA from oxidative damage, reduce the expression of cancer-promoting genes, and inhibit tumor growth. Research has shown that women who consume high amounts of EVOO have a reduced risk of developing hormone receptor-positive breast cancer.
**Fruits and Vegetables: A Source of Vitamins and Bioactive Compounds**. The Mediterranean diet emphasizes a high intake of colorful fruits and vegetables, which provide essential vitamins, minerals, and phytochemicals such as flavonoids, carotenoids, and polyphenols. These compounds have strong antioxidant and anti-inflammatory properties, neutralizing free radicals that can cause DNA mutations, leading to cancer. Additionally, cruciferous vegetables like broccoli, cabbage, and kale have sulforaphane, which has been shown to inhibit cancer cell proliferation and promote apoptosis (programmed cell death) in breast cancer cells.
**Whole Grains and Legumes: High in Fiber and Phytoestrogens**. Whole grains (such as oats, quinoa, and whole wheat) and legumes (such as lentils, chickpeas, and beans) are excellent sources of **fiber**, which aids in digestion and plays a key role in regulating estrogen levels in the body. Excess estrogen is a risk factor for hormone-dependent breast cancers, and fiber helps bind estrogen in the gut, promoting its excretion and reducing its circulating levels. Moreover, legumes have phytoestrogens, plant-derived compounds that mimic estrogen but have protective effects against hormone-related cancers.
**Omega-3 Fatty Acids: Anti-Inflammatory and Tumor-Suppressing Properties**. The Mediterranean diet includes fatty fish, like salmon, sardines, and mackerel, which are rich in omega-3 fatty acids. These essential fatty acids have been shown to reduce chronic inflammation, inhibit cancer cell proliferation, and prevent angiogenesis (the formation of new blood vessels that supply tumors). A balanced intake of omega-3 and omega-6 fatty acids is crucial, as an excess of omega-6 fatty acids (found in processed foods and vegetable oils) can promote inflammation and tumor growth.
**Nuts and Seeds: A Source of Healthy Fats and Polyphenols**. Almonds, walnuts, flaxseeds, and chia seeds are staple ingredients in the Mediterranean diet. They provide healthy fats, fiber, and polyphenols, which contribute to reducing inflammation and oxidative stress. Walnuts, for instance, have ellagic acid, which has been shown to suppress breast cancer cell growth, while flaxseeds are rich in **lignans**, a type of phytoestrogen that helps regulate estrogen metabolism.
**Red Wine: Resveratrol and Its Anti-Cancer Potential**. In moderation, red wine is consumed in the Mediterranean diet and is a natural source of resveratrol, a polyphenol with antioxidant, anti-inflammatory, and anti-cancer properties. Resveratrol has been found to induce apoptosis in breast cancer cells, inhibit their proliferation, and suppress tumor growth. However, it is important to note that excessive alcohol consumption is associated with an increased risk of breast cancer, so intake should be limited to small amounts (e.g., one glass per day for women).
**Reduced Consumption of Processed and Red Meats.** Unlike Western diets, which often include high amounts of red and processed meats, the Mediterranean diet prioritizes lean protein sources, such as fish and plant-based proteins. Processed and red meats have compounds like heterocyclic amines and polycyclic aromatic hydrocarbons, which are formed during high-temperature cooking and have been linked to cancer development. Reducing consumption of these meats can lower exposure to these carcinogenic compounds.

## Abbreviations

The following abbreviations are used in this manuscript:

MedDiet	Mediterranean Diet
ROS	Reactive Oxygen Species
PPs	Polyphenols
TNBC	Triple-Negative Breast Cancer
